# Massive Neurilemoma of the Hard Plate in Which Preoperative Diagnosis Was Difficult

**DOI:** 10.1155/2015/638025

**Published:** 2015-08-02

**Authors:** Masanori Kudoh, Hiroyuki Harada, Koshi Matsumoto, Yuriko Sato, Ken Omura, Yoshimasa Ishii

**Affiliations:** ^1^Division of Oral and Maxillofacial Surgery, Ebina General Hospital, 1320 Kawaraguchi, Ebina City, Kanagawa 243-0433, Japan; ^2^Oral and Maxillofacial Surgery, Department of Oral Restitution, Division of Oral Health Sciences, Graduate School, Tokyo Medical and Dental University, Japan; ^3^Division of Diagnostic Pathology, Ebina General Hospital, Japan; ^4^Division of Oral and Maxillofacial Surgery, General Tokyo Hospital, Japan

## Abstract

The patient was an 84-year-old man who was referred to our hospital in mid-December 2012 for a close examination of a mass arising from the left side of the hard palate that was found by a local dentist. The initial examination revealed the presence of a 3.0-cm elastic soft, dome-shaped mass in the left hard palate. CE-CT showed a lesion of size 1.8 × 1.4 cm in the right hard palate, which extended upward and invaded the nasal cavity. The mass was a solid tumor associated with resorption of surrounding bone and expansion of the greater palatine canal. CE-MRI indicated that the mass extended upward and invaded the nasal cavity, and the mass showed hypointensity on T1-weighted images, hyperintensity on T2-weighted images, and an irregular margin with internal enhancement. Abnormal uptake of FDG on PET-CT (SUVmax = 5.2) was observed in the left hard palate. The biopsy site lesion rapidly increased in size and biopsy was performed again in January 2013 due to suspicion of a malignant tumor. The histopathological diagnosis was a suspected malignant neurogenic tumor. Therefore, the patient underwent partial maxillectomy and a split-thickness skin graft in late February 2013. No recurrence was noted 29 months after the operation.

## 1. Introduction

Neurilemoma is a benign tumor derived from Schwann cells or perineural fibroblasts. Most cases develop in the extremities or under the skin in the head and neck region. The tumor is most common in the buccal area and under the temporal fossa in the head and neck region and less common in the oral region, which accounts for only 0.02% of cases [1, 2]. Intraorally, the tongue is the most common site, and the tumor is less common in the buccal mucosa, floor of the mouth, gingiva, and palate [3, 4]. Here, we describe an extremely rare case of massive neurilemoma of the hard palate, in which the tumor was thought to be derived from the greater palatine nerve.

## 2. Case Presentation

The patient was an 84-year-old man who was referred to our hospital in mid-December 2012 for a close examination of a mass arising from the right side of the hard palate, which was found by a local dentist in early December 2012. He had prediabetes and was receiving diet therapy. His other medical history included HBV carrier, TPHA positive, and prostatic hyperplasia, which was under treatment with oral drugs by a local physician. He was a medium-sized man and his nutritional status was good. His family history was unremarkable.

**Figure 1:**
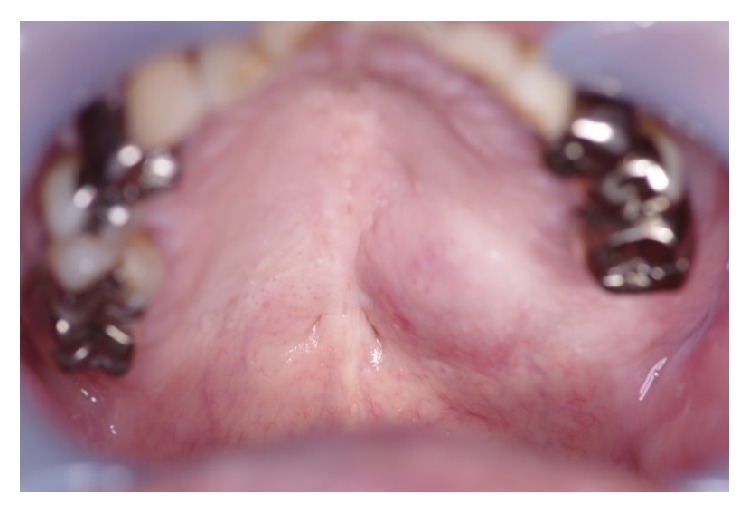
Intraoral findings at the first visit.

**Figure 2:**
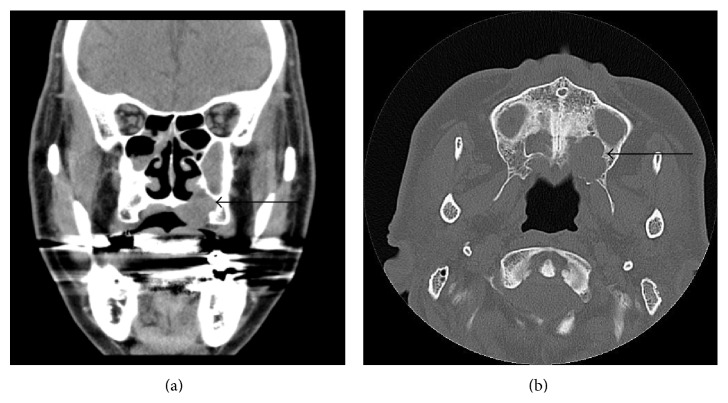
CT images. (a) Frontal plane CE-CT image showing the presence of a 1.8 × 1.4 cm mass in the left hard palate, which extended upward and invaded the nasal cavity (arrow). (b) Horizontal image showing hyperplasia localized in bone (arrow) observed upward and backward to the maxillary sinus. No bone destruction was observed.

**Figure 3:**
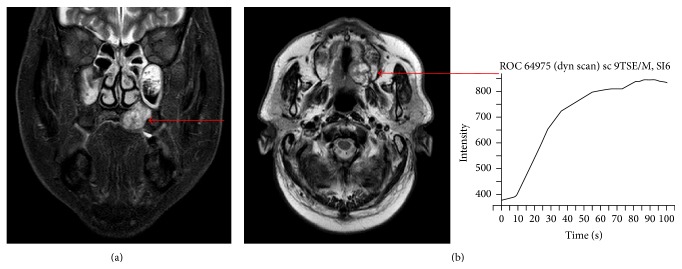
MR images. (a) Frontal plane section. (b) Horizontal section. CE-MRI showed that the mass extended upward and invaded the nasal cavity. The mass showed hypointensity on T1-weighted images, hyperintensity on T2-weighted images, and an irregular margin with internal enhancement. Dynamic MRI revealed a plateau type lesion that was suspected to be a malignant tumor.

**Figure 4:**
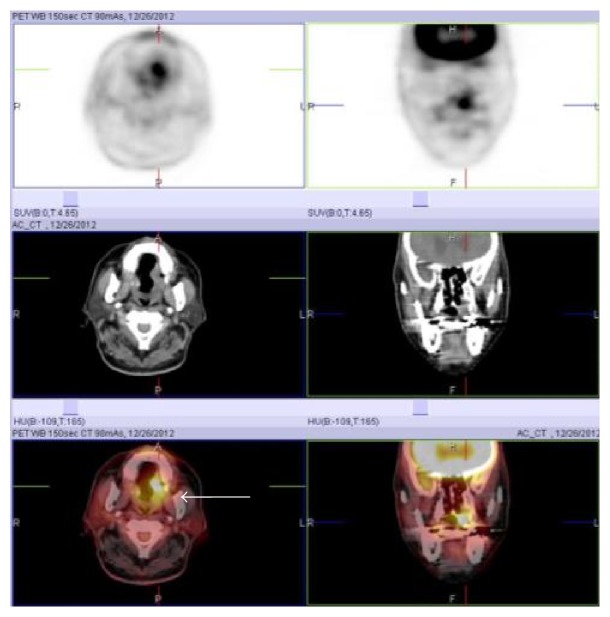
PET-CT image showing abnormal uptake of FDG (SUVmax = 5.2) in the left hard palate.

Initial oral findings showed a 3.0-cm elastic soft, dome-shaped mass in the right hard palate (Figure 1). CE-CT showed a lesion of size 1.8 × 1.4 cm in the right hard palate, which extended upward and invaded the nasal cavity (Figure 2(a)). The mass was a solid tumor associated with resorption of surrounding bone and an enlarged greater palatine canal (Figure 2(b)). CE-MRI indicated that the mass extended upward and invaded the nasal cavity, and the mass showed hypointensity on T1-weighted images, hyperintensity on T2-weighted images, and an irregular margin with internal enhancement (Figure 3(a)). Dynamic MRI revealed a plateau type lesion that was suspected to be a malignant tumor (Figure 3(b)). Abnormal uptake of FDG in PET-CT (SUVmax = 5.2) was observed in the left hard palate (Figure 4). Based on these findings, the patient was diagnosed with a malignant tumor in the left hard palate.

**Figure 5:**
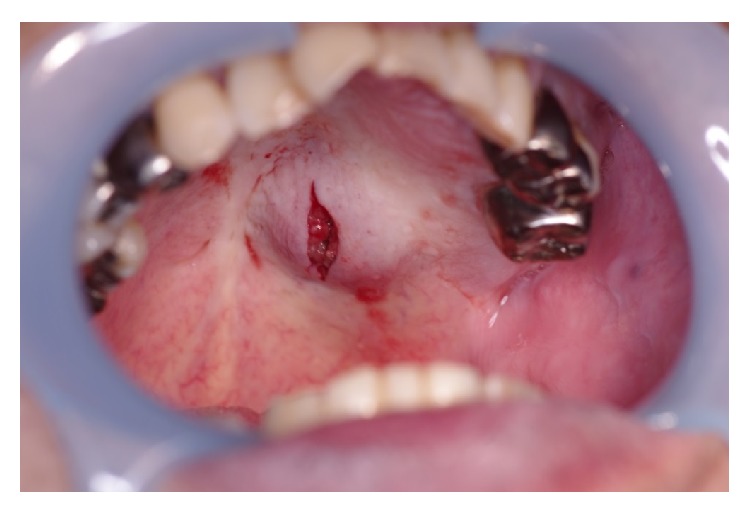
Intraoral findings after the first biopsy.

**Figure 6:**
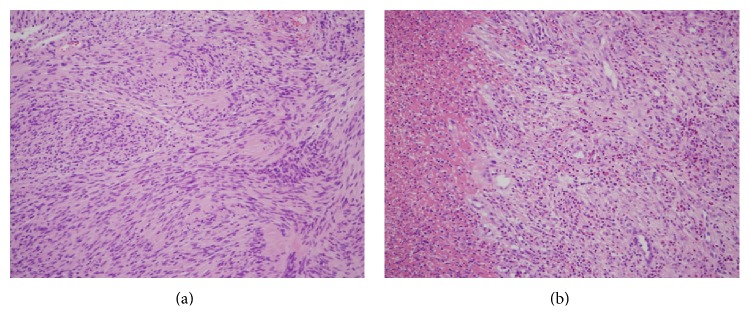
Histopathological image (first biopsy: a, b: H-E ×200). Eosinophilic spindle cells with a round-like or oval nucleus were proliferating in the lamina propria. There were rough myxoma-like lesions and those with a high cell density palisading arrangement depending on the location, and abundant inner vascularity.

**Figure 7:**
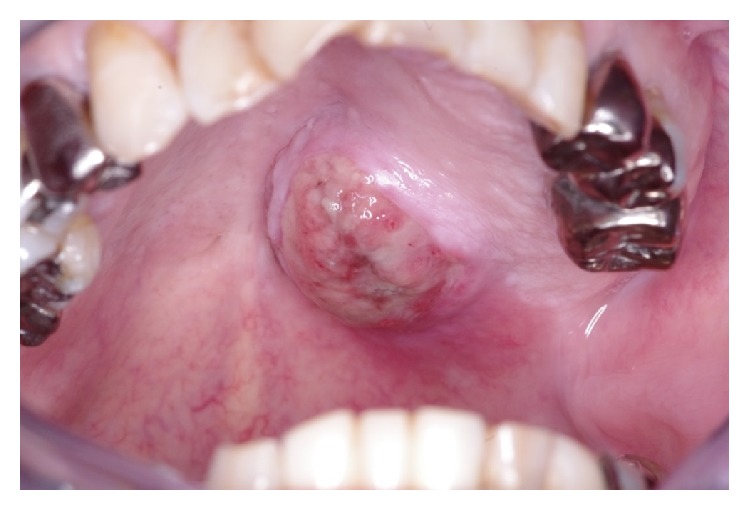
After re-biopsy, the lesion size rapidly increased and a malignant tumor was strongly suspected.

**Figure 8:**
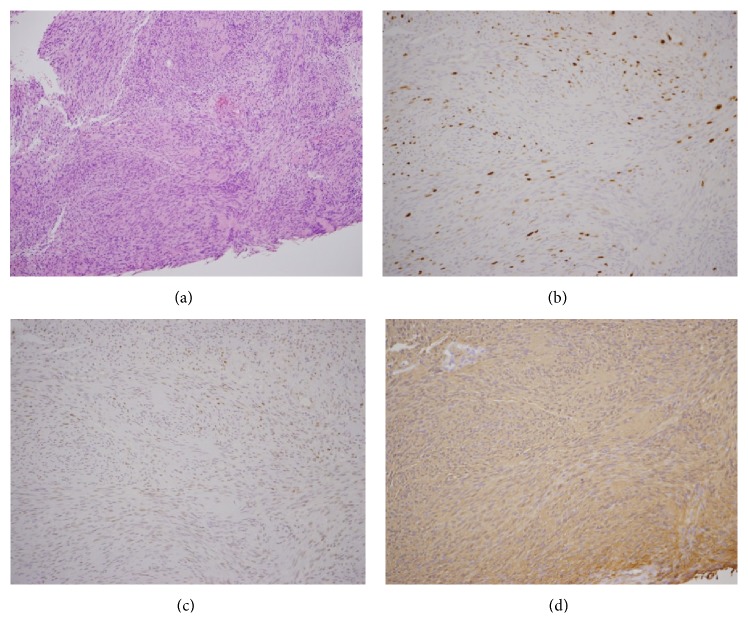
Histopathological image (rebiopsy: a, b: H-E ×100, Ki-67 ×200). Tumor cells containing atypical glands and a spindle nucleus, with fibrous tissues in the submucosa proliferating in a fascicular pattern. Mitosis and proliferation of atypical cells were present, and immunostaining showed a Ki-67 positive rate exceeding 5%. Histopathological image (rebiopsy: c, d: S-100 ×200, p63 ×200). The tumor cells were S-100 positive and strongly p63 positive.

Biopsy was performed twice and the tumor was confirmed histopathologically to be neurilemoma (Figures 5, 6(a), and 6(b)). However, the biopsy site lesion showed aggressive rapid growth (Figure 7), and therefore rebiopsy was performed in January 2013 due to suspicion of a malignant tumor. Histopathological specimens indicated that the lesion consisted of tumor cells containing atypical glands and a spindle nucleus, with fibrous tissues in the submucosa, which proliferated in a fascicular pattern. Mitoses and proliferation of atypical cells were also present (Figure 8(a)). In immunostaining, the Ki-67 positive rate exceeded 5% and the tumor cells were S-100 positive and strongly p63 positive. Based on these findings and the disease progression, the lesion was histopathologically diagnosed as a suspected malignant neurogenic tumor (Figures 8(b), 8(c), and 8(d)).

**Figure 9:**
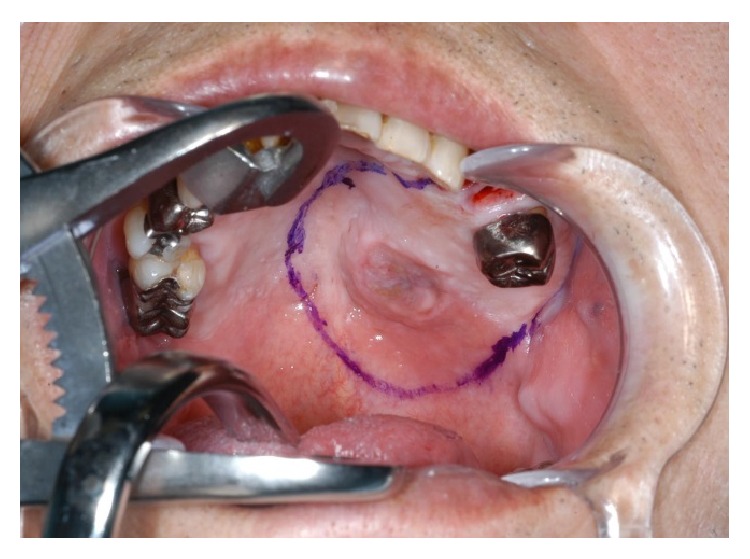
Intraoperative findings.

**Figure 10:**
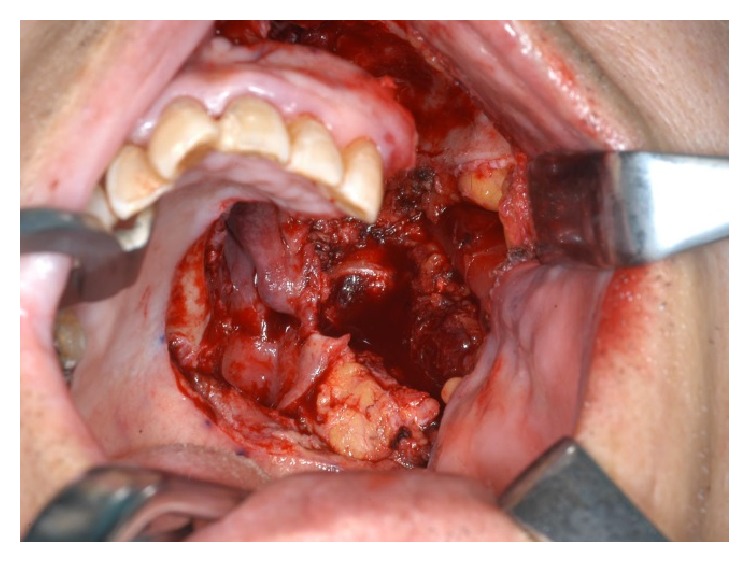
After resection of the tumor.

**Figure 11:**
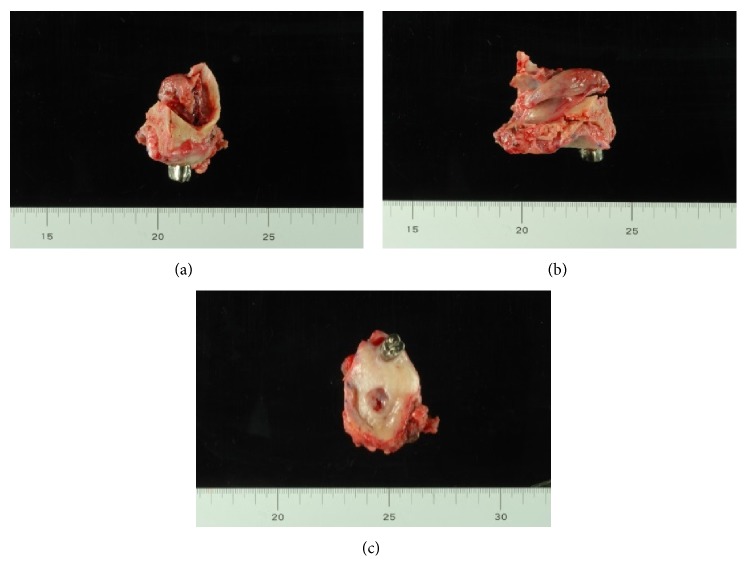
Resected specimens. (a) Frontal plane section. (b) Sagittal section. (c) Horizontal section.

The patient underwent partial maxillectomy and a split-thickness skin graft under general anesthesia in late February 2013 in the Division of Oral and Maxillofacial Surgery, Tokyo Medical and Dental University. The margin was located 0.8 cm from the tumor on the palate (Figure 9). The anterior wall of the maxilla was cut at the level of the middle meatus to open the maxillary sinus. The maxillary sinus membrane was edematously thickened, but there was no tumor exposure in the sinus. The maxillary sinus membrane was detached and collected on the floor of the sinus, and subsequently osteotomy was performed from the lateral wall to the posterior wall of the maxilla at almost the same level. Then, the alveolar process was cut in the left maxillary first premolar area and the palate bone was separated at almost the same level as the incision line of the membrane. The maxilla was downfractured to separate the pterygoid process and cut the internal pterygoid muscle, in order to isolate them with the maxilla (Figures 10, 11(a), 11(b), and 11(c)). Collected STS was grafted in the pterygoid region. After the sulcular incision in the anterior lip was sutured, tetracycline hydrochloride carboxymethyl cellulose ointment dressing gauze was put into the surgical cavity. Finally, the wound was closed and surgery was completed.

**Figure 12:**
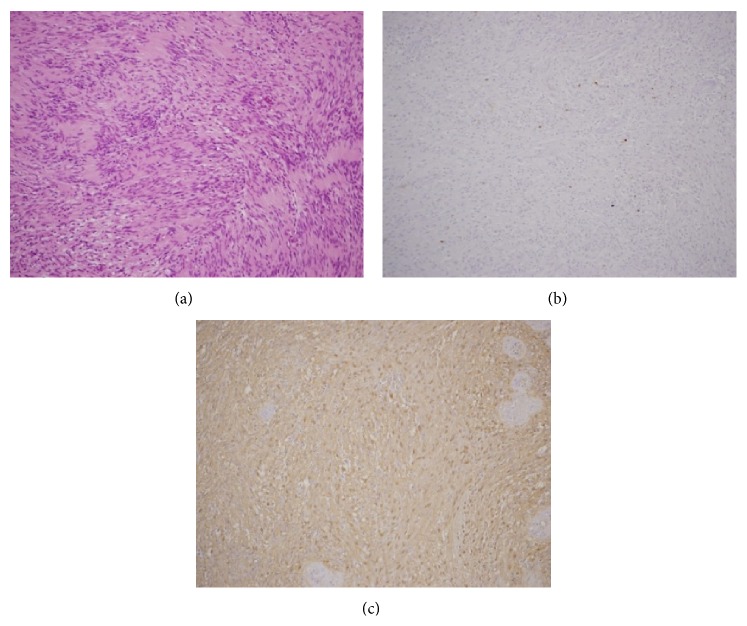
Histopathological images. (a) H-E staining ×200. (b) Ki-76 ×200. (c) S-100 ×200.

**Figure 13:**
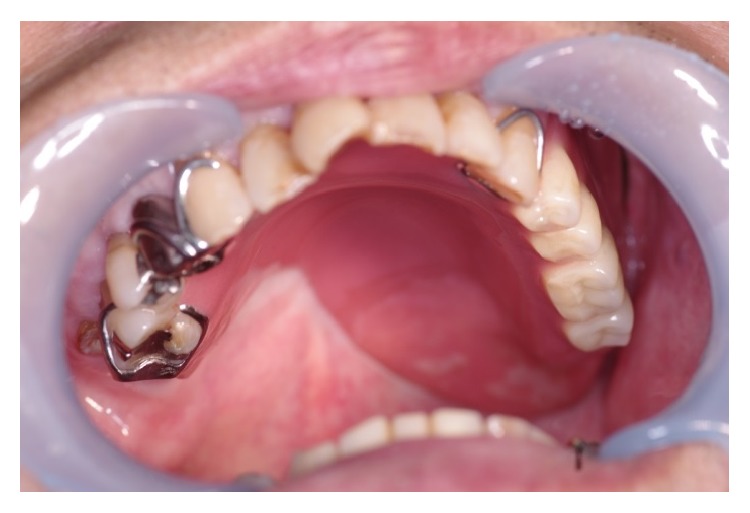
After placement of a denture for a defective maxilla.

Histopathologically, the surgical specimen showed disordered proliferation of spindle-shaped cells, which consisted of mainly Antoni A type showing palisading features and a small amount of Antoni B type in a slightly edematous substrate. In immunostaining, the cells were S-100 positive, the Ki-67 positive rate was approximately 1%, and neither atypical nor nuclear divisions were observed (Figure 12). Histopathology showed neurilemoma. Currently, at 29 months postoperatively, the patient is in excellent condition without recurrence. He wears a denture for a defective maxilla, and his velopharyngeal, masticatory, and articulatory functions have recovered (Figure 13).

## 3. Discussion

Neurilemoma is a benign tumor derived from Schwann cells or perineural fibroblasts. The tumor was named by Verocay [5] in 1910 based on its involvement with proliferation of ectodermal Schwann cells. There are many hypotheses regarding the mechanism of onset, including an ectodermal tumor derived from Schwann cells [5] and a mesodermal tumor derived from perineural tissues [6, 7]. Recent electron microscopy, tissue culture, and immunohistochemical analyses strongly suggest that the former hypothesis may be correct.

In immunohistochemistry using a S-100 protein antibody, most of the tumor cells in our patient were S-100-positive. S-100 is expressed in peripheral Schwann cells [8], and Schwann cells are considered to be a tumor marker. An electron micrograph showed disordered proliferation of spindle-shaped cells, which is specific to Schwann cells with a palisading pattern. These findings strongly suggested an ectodermal tumor derived from Schwann cells, as proposed by Verocay [5]. Intraoperative findings show that the primary lesion in cases in the hard palate originates from coalescence of the greater palatine nerve, lesser palatine nerve, and nasopalatine nerve [9]. In our case, imaging and intraoperative findings suggested that the probable origin of the primary lesion was the greater palatine nerve.

Intraorally, neurilemoma is less likely to occur in the palate and only 42 such cases have been reported in Japan [10]. The age of the patients ranged from 4 to 79 years old and the mean age was 30.3 years old. Sasaki et al. [11] found that the peak age of onset was 10 to 20 years old, the mean age was 25.6 years old, the incidence was slightly higher in females (56.1%), and the tumor diameter was likely to be 1 to 3 cm, with only 3 patients (7.3%) having a tumor diameter >4 cm [11]. The highest incidence of neurilemoma in the oral cavity occurs in the tongue, at rates of 45.2% (in 157 patients who developed neurilemoma in the oral and maxillofacial region) in Gallo et al. [12] and 35 to 45% in Yamada et al. [13]. In Gallo et al. [12], the tumor location was the tongue in 71 patients, followed by the buccal mucosa in 21, mandible in 18, palate in 12, gingiva in 7, lips in 7, oral vestibule in 5, and maxilla in 2. The incidence of the tumor in the hard palate is only 7–9% [12, 13].

Neurilemoma in the oral and maxillofacial region is generally a smooth surfaced, elastic soft, and sharply circumscribed localized tumor mass that is painless [14] and is characterized by slow and painless growth [14]. However, some cases of neurilemoma in the hard palate (5 patients, including our patient: 4 females and 1 male) [15–18] have been found to be hemorrhagic and rapidly growing granuloma-like tumors, which are difficult to differentiate from malignant tumors. Since the initial clinical diagnosis in the present case was a malignant tumor in the right hard palate, biopsy was performed twice and both results indicated a neurilemoma. However, the biopsy site lesion rapidly increased in size, and therefore we suspected a malignant neurogenic tumor and rebiopsy was performed. The findings from this biopsy and the disease progression led to a histopathological diagnosis of a suspected malignant neurogenic tumor.

For an asymptomatic, slowly growing neurilemoma in the hard palate, as found in our patient, surgical invasion is likely to produce and cause rapid growth of granulation tissue, and some cases may have an abnormally regenerated epithelium at the biopsy site. Such lesions are likely to be difficult to differentiate from malignant tumors. A successful preoperative diagnosis in this case was achieved by (1) monitoring of rapid growth, proliferation, and formation of granuloma, (2) CE-CT showing progression of the tumor and bone destruction in the palate and nasal cavity, exclusion and resorption of the surrounding bone, and expansion of the greater palatine canal, (3) CE-MRI showing hypervascularity of the tumor with internal enhancement and an irregular margin, (4) PET-CT showing a primary lesion with abnormal uptake of FDG, and (5) diagnosis of a malignant neurogenic tumor by rebiopsy. A malignant neurogenic tumor was suspected in histopathological diagnosis based on the rebiopsy result because the aggressive granulation tissues and tumor cells were positive for Ki-67 or p53 and because aggressive rapid tumor growth and expansion seen on imaging suggested low grade or borderline malignancy.

There are several reports of cases with aggressive rapid tumor growth and expansion which is considered to be a malignancy, after biopsy or isolation of neurilemoma [15–18]. Currently, at 29 months after surgery, our patient wears a denture for a defective maxilla and is in excellent condition without recurrence, with improved QOL and recovery of velopharyngeal, masticatory, and articulatory functions. However, postoperative recurrence or malignant transformation can occur in cases of neurilemoma of the palate [19–21] and close long-term follow-up will continue for our patient.

## 4. Conclusion

Here, we described an extremely rare case of massive neurilemoma of the hard palate, in which preoperative diagnosis was difficult.
